# A review of herb-induced liver injury in mainland china

**DOI:** 10.3389/fphar.2022.813073

**Published:** 2022-10-11

**Authors:** Yan Yang, Fei-Lin Ge, Jin-Fa Tang, Shuang-Lin Qin, Rui Zeng, Meng-Lin Yao, Xiao-He Xiao, Zhao-Fang Bai, Cheng-Lin Tang

**Affiliations:** ^1^ College of Traditional Chinese Medicine, Chongqing Medical University, Chongqing, China; ^2^ Department of Chinese Medicine, The First Affiliated Hospital of Zhengzhou University, Zhengzhou, China; ^3^ Department of Pharmacy, The First Affiliated Hospital of Henan University of Chinese Medicine, Zhengzhou, China; ^4^ School of Pharmacy, Xianning Medical College, Hubei University of Science and Technology, Xianning, China; ^5^ School of Integrated Traditional Chinese and Western Medicine, Southwest Medical University, Luzhou, China; ^6^ Senior Department of Hepatology, The Fifth Medical Center of PLA General Hospital, Beijing, China

**Keywords:** pharmacoepidemiology, risk factors, risk prevention and control, herb, pharmacovigilance

## Abstract

Traditional medicines have greatly contributed to people’s health worldwide. However, in recent years, the frequent occurrence of herb-induced liver injury (HILI) has raised public concerns regarding the safety of herbs. HILI not only severely impacts public health, thus increasing its medical burden, but also consumes medical resources. However, the pharmacoepidemiology and risk factors of HILI are still unclear due to the complexity of herbs (medication theory, drug composition, dual properties of drugs and food, etc.). China is the country with the most extensive use of herbs and cases of HILI worldwide. The safety profile of herbs (especially with respect to HILI) has also affected the use of herbs internationally. Therefore, this review focuses on the epidemic situation of HILI in mainland China to compile its characteristics, while focusing on the three main aspects of patients, drugs, and unreasonable prescriptions to explore the potential risk factors. Our objective was to provide a reference for HILI pharmacovigilance and risk prevention and control and contribute to Chinese knowledge of the realisation of the “Medication without Harm” global safe medication strategic goal of the World Health Organization.

## 1 Introduction

For thousands of years, traditional medicine has made considerable contributions to people’s health worldwide. Traditional Chinese medicine (TCM), a complementary and alternative system of medicine, has continued to play a major role in human health. The discovery of artemisinin for the treatment of malaria was honoured with the Nobel Prize in medicine (Li et al., 2021). However, in recent years, there have been frequent occurrences of herb-induced liver injury (HILI) caused by Polygoni Multiflori Radix (PM), Psoraleae Fructus (PF), and aristolochic acid (AA), among others, arousing public concern about the safety of herbs especially regarding liver injury (Yu et al., 2015; Ge et al., 2019a; Ge et al., 2019b; Ballotin et al., 2021; Ge et al., 2021; Teschke et al., 2021). HILI not only severely impacts public health, thus increasing its medical burden, but also consumes medical resources (Ge et al., 2018). What’s worse, owing to the complexity of HILI, its pharmacoepidemiology and risk factors remain unclear, and there is no reliable evidence for risk control.

China is one of the world’s largest consumers of traditional drugs (TD), and the country with the highest incidence of HILI. The safety of TCM, especially regarding HILI, has a large impact on global medication safety, including TD (Xiao et al., 2021a; Xiao et al., 2021b). Therefore, we reviewed the pharmacoepidemiological characteristics of HILI in China, including the epidemic trend, the proportion of HILI among cases with drug-induced liver injury (DILI), and distribution of major HILI drugs. Based on this, we focused on potential risk factors in terms of patients, drugs, and unreasonable prescriptions. This review aimed to provide a reference for China’s HILI pharmacovigilance and risk prevention and control and contribute to Chinese knowledge of the realisation of the “Medication without Harm” global safe medication strategic goal of the World Health Organization (WHO).

## 2 Pharmacoepidemiology

### 2.1 Incidence and prevalence of herb-induced liver injury

In the epidemiological studies (retrospective observation) on DILI/HILI, the incidence has been a difficult and controversial topic. Currently, the internationally recognised incidence of DILI is 19.1/100,000 and 13.9/100,000 people (Dominique, 2002; Björnsson et al., 2013). However, the incidence of DILI/HILI in China is still unclear, and two recent studies (retrospective observation) with large sample sizes estimated the incidence of DILI in China to be 23.8/100,000 and 320/100,000 people respectively, which is higher than that in the West (Shen et al., 2019; Kong et al., 2021). The incidence of HILI was calculated as the number of people who took herbs and developed HILI thereafter. However, it is difficult to obtain such data accurately in practice. Herbs are not only used as medicine but also as food, which makes it difficult to accurately assess the number of people who consume herbs and dietary supplements (HDS). Herbs are frequently combined with conventional medicine (CM) in clinics, making it difficult to determine whether DILI can be attributed to HILI (Ge et al., 2018; Ge et al., 2019a). In addition, owing to the large population of China and the unequal development between different regions, it is difficult to conduct epidemiological studies on HILI based on the national population or a well-represented multi-center population, which markedly restricts high-quality epidemiological studies on HILI, especially concerning the incidence of HILI in China.

The frequency of HILI is unclear, thus its prevalence remains uncertain. We consulted Chinese (CNKI/VIP/WanFang) and English literature (Pubmed) databaes from database establishment until Sep. 2021. We searched for “Traditional Chinese Medicine” or “herb” and “liver injury”, or “HILI”, which produced an increasing trend of HILI-related studies. The frequency of HILI and the proportion of HILI among DILI cases presented an increasing trend in an adverse drug reaction (ADR) analysis by the National Medical Products Administration of China (NMPA) between 2012 and 2016. Due to bias, neither the literature search nor ADR reports of the NMPA directly reflect the epidemic trend of HILI. However, this increasing trend reflects the importance of HILI prevention and control.

### 2.2 Proportion of herb-induced liver injury among drug-induced liver injury cases

The proportion of HILI among DILI cases was derived from a retrospective observational study. In Asia, especially in China, herbs are frequently the main cause of DILI, accounting for approximately 30% (Yu et al., 2015; Devarbhavi et al., 2021). In China, a nationwide study that included 25,927 DILI cases indicated that 26.81% of the cases were associated with TCM/HDS products (Shen et al., 2019). Two literature-based epidemiological studies on DILI in mainland China (database search period - December 2019/January 1998–December 2018 showed that HILI accounted for 30% and 30.28% of the cases, respectively (Wang J. et al., 2019a; Yang et al., 2020). A prospective study conducted in South Korea indicated that herbs were associated with 27.5% of DILI cases (Suk et al., 2012). In Japan, HDS were associated with 15% of DILI cases in a prospective study (Aiso et al., 2019).

**FIGURE 1:**
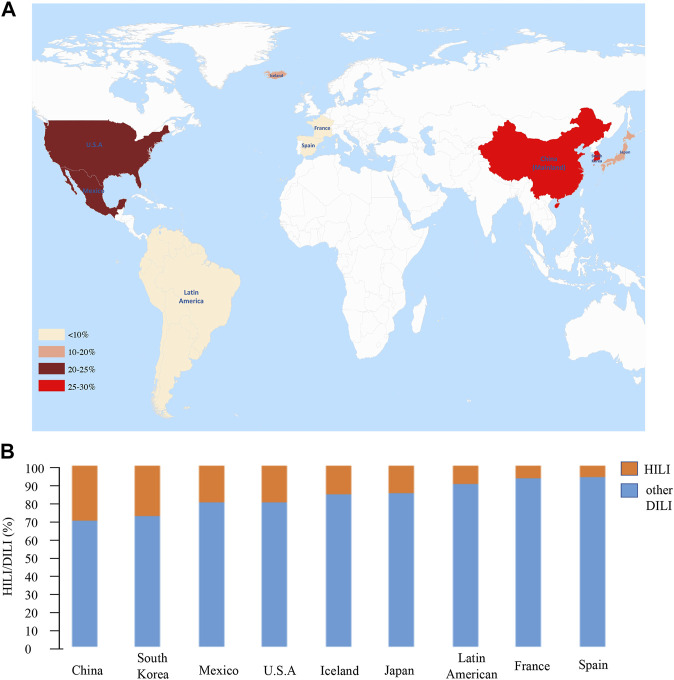
The proportion of HILI in large-sample DILI studies. **(A)** Heat map. The redder the colour, the higher the proportion of HILI among DILI cases. **(B)** Histogram. Orange indicates the proportion of HILI, blue indicates the proportion of other DILI except for HILI, and more orange represents the more proportion of HILI in DILI.

In the West, HILI accounts for an increasing proportion of DILI cases. The incidence of HILI in the Spanish DILI registry ranged from 2% in 1998 to 6% in 2016 (Andrade et al., 2018). The incidence of liver injury caused by HDS ranged from 7% to 20% in Mexico (Liu et al., 2016). Data from the DILI network (DILIN) registry indicated that the proportion of HDS DILI cases was 7% in France and 20% in the United States (Dominique, 2002; Navarro et al., 2014). A nationwide study from Iceland demonstrated that 16% of DILI cases are attributable to HDS (Björnsson et al., 2013). In Latin America, preliminary data from the LATIN DILIN showed that 10% of acute liver injury cases were attributed to HDS (Andrade et al., 2018) (Figure 1).

TCM has been used in China for thousands of years, and the Chinese population generally trust TCM, hence, the herbal market is vast. All drug can exert toxic side effects, for example, acetaminophen (APAP) has a wide range of clinical applications as a common analgesic drug, but it is also the most common drug associated with DILI (Yu et al., 2015). Therefore, the widespread use of herbs in China has increased the risk of HILI to a certain extent. China has the highest number of HILI cases, and the underlying reasons are complex and diverse. In addition to the vast extent of consumers, three reasons also includes many other factors such as physical factors. Further HILI risk factors in China are discussed in detail below.

In recent years, the global consumption of HDS products has rapidly increased. In addition to China, Western countries also use herbs and related products widely, resulting in an increasing number of HILI. In China, herbs are regulated through the NMPA. However, in most other countries, herbs are not included in drug regulatory systems, therefore, the quality of HDS products is not guaranteed, and it is difficult to identify the exact ingredients of herbal preparations. The ingredients of many herbal products that are currently in use are complex and frequently unclear, particularly in multi-compound products. Owing to a lack of strict standards and supervision, herbal concoctions may be produced through substitution with various plant species. They may be adulterated with heavy metals, pesticides, herbicides, and microorganisms, which may also contribute to hepatotoxicity. Thus, even “safe” herbal products may be contaminated with toxic compounds that cause hepatotoxicity (Chalasani et al., 2021). In addition, HDS are generally believed in the West to be produced from natural and non-toxic plant sources, which may be an important risk factor for HILI (Chalasani et al., 2015; Navarro et al., 2017).

### 2.3 Distribution of suspected herbs presumably associated with herb-induced liver injury

Clinical evaluation of HILI has always been a difficult problem owing to numerous factors and complex clinical applications. Based on different data sources, including the NMPA (CFDA., 2014), the Liver Tox website (Hoofnagle et al., 2013), case series reports, case reports, animal experiments, and *in vitro* experiments, etc., we reviewed common herbs related to HILI to provide a reference and basis for clinical and experimental reserach. Commonly herbs associated with HILI include PM (Tu et al., 2015; Wang et al., 2016; Zhang B. et al., 2017a; Tu et al., 2019a; Tu et al., 2019b; Li et al., 2019; Zhang et al., 2019), PF(Wu et al., 2017; Wang et al., 2018b; Qin et al., 2021), Epimedii Folium (EF) (Zhang et al., 2018; Gao et al., 2020; Wang et al., 2020; Gao Y. et al., 2021a), Dictamni Cortex (DC) (Huang et al., 2017; Shi et al., 2019; Shi, 2020), Gynura Segetum (GS) (Gou et al., 2017; Ding and Xiong, 2018; Li et al., 2018; Tong et al., 2021), Rhizoma Dioscoreae Bulbiferae (RDB) (Wang and Liao, 2018a; Li C. Y. et al., 2020a; Hou et al., 2020; Chen D. Y. et al., 2021a), Senecionis Scandentis Hebra (SSH) (Xiong et al., 2020; Jiang et al., 2021; Ye et al., 2021), Tripterygii Radix (TR) (Yao et al., 2016; Zhu C. S. et al., 2018a; Zhou et al., 2018; Zhou et al., 2018; Song et al., 2019; Liu et al., 2021), Bruceae Fructus (BF) (Ma and Chen, 2014; Wu et al., 2018), Rhei Radix Et Rhizoma (RRR) (Wang et al., 2011; Yao et al., 2020; Huo et al., 2021). The NMPA has warned of a risk of liver injury in TCM preparations including Yangxue Shengfa capsules (CFDA., 2014), Shouwu tablet (CFDA., 2014), Shouwu pill (CFDA., 2014), Shouwu Yanshou tablet (CFDA., 2014), Shouwu Yanshou granules (CFDA., 2014), Xianling Gubao capsule (Li J. et al., 2020b), Zhuanggu Guanjie pill (Tang et al., 2018; Xiong et al., 2019), Gukang capsule (Zhu L. et al., 2018b; Liu et al., 2019), Qingdai pill (Fang et al., 2004), Zhixue capsule (Li P. et al., 2017a), and Baishi pill (Wu et al., 2012).

With regard to the list of herbs suspected to be associated with HILI (Table 1), TR and GS are highly toxic and their toxic effects have been demonstrated in TCM literature and modern research. However, herbs that mainly enhance immunity and are safe according to TCM standards such as PM, PF and EF, may also cause HILI (Gao et al., 2020; Ge et al., 2021; Li et al., 2019). Owing to this phenomenon, some scholars believe that liver injury caused by such herbs enhancing immunity may be attributable to idiosyncratic HILI (IHILI). Further studies (retrospective and prospective observational studies) have found that people with high levels of immune gene expression, cytokines, and biological metabolites have a significantly higher risk of HILI when taking such herbs (Tang et al., 2018; Li et al., 2019). IHILI differs from intrinsic HILI (InHILI), and there is no obvious correlation between the dosage of the medicine and hepatotoxicity. IHILI is mainly associated with physical factors and is delayed, insidious, accidental, specific and indirect. Therefore, it is difficult to prevent and control IHILI caused by herbs simply by adjusting the dosage, which significantly increases the difficulty of risk prevention and control.

**TABLE 1:** The list of suspected herbs related to HILI.

HILI reports							Toxic compounds	Number of herbal preparations	Type
HILI related to herbs	NPMA	Liver tox	Case series reports	Case reports	Animal experi-ments	Vitro experi-ments			
Polygoni Multiflori Radix	+	+	+	+	+	+	cis-stilbene glycoside emodin-8-glucoside	398	IHILI
Epimedii Folium	−	−	+	+	+	+	icariside II	349	IHILI
							icariside I		
Psoraleae Fructus	−	+	+	+	+	+	Bavachin, Psoralidin	225	IHILI
Dictamni Cortex	−	−	+	+	+	−	isomaculosidine	49	IHILI
Gynura Segetum	−	−	+	+	+	−	pyrrolizidine alkaloids	1	InHILI
Rhizoma Dioscoreae Bulbiferae	−	−	+	+	+	+	diosbulbin B	9	InHILI
							8- epifloxacin acetate		
Senecionis Scandentis Hebra	−	−	−	+	+	+	senecionine	26	InHILI
							adonifoline		
Tripterygii Radix	+	−	+	+	+	+	Tripterygium glycosides triptolide	6	InHILI
Bruceae Fructus	−	−	+	+	−	−	oleum fructus bruceae	5	InHILI
Rhei Radix ET Rhizoma	−	−	−	−	+	+	emodin	807	InHILI
							emodin-8-glucoside		

NMPA, National Medical Products Administration of China; IHILI, idiosyncratic HILI; InHILI, intrinsic HILI.

### 2.4 Prevalence of herb-induced liver injury in Hong Kong and Taiwan, China

As important parts of China, HongKong and Taiwan may provide referential data for the epidemiology of HILI. A recent retrospective epidemiological survey of HILI in Taiwan showed that HILI accounted for 22.0% of DILI cases. In terms of general demographic data, sex:male/female = 1.26/1 (*p* > 0.05), age: 57.3 ± 15.2. HILI-associated drugs primarily included Xiao Chai Hu Tang, Long Dan Xie Gan Tang, Cang Er San, Dysosma pleiantha, Snake gall bladder, Tripterygium wilfordii, and PM. Compared to the DILI group, the HILI group had higher initial serum alanine aminotransferase, alkaline phosphatase (ALP), peak ALP, and bilirubin levels, and higher rates of jaundice, ascites, encephalopathy, coagulopathy, sepsis, and acute liver failure. In addition, the HILI group had a higher mortality rate than the DILI group (12.6% vs. 8.0%, *p* = 0.016). Hepatitis B carrier status, elevated baseline liver biochemical tests results and the use of crude herbs (without processing) were associated with an increased risk of HILI-related mortality (Huang et al., 2021). There is no epidemiological study of HILI in Hong Kong, however, case reports on HILI in Hong Kong involved PF and PM (Lin et al., 2019).

## 3 Risk factors of herb-induced liver injury

### 3.1 Patient factors

HILI can be classified into IHILI and InHILI. InHILI is predictable, and closely related to the drug dosage; it shows a short incubation period. In contrast, IHILI is unpredictable and frequently not correlated with drug dosage. In addition, IHILI shows marked individual differences, and animal experiments are difficult to replicate. The following section mainly discusses the risk factors of IHILI.

#### 3.1.1 Idiosyncratic factors

Idiosyncratic factors include immune and genetic idiosyncrasies. The risk of liver injury is significantly higher in patients with specific immune characteristics taking certain drugs than that in other patients. Studies (retrospective observation) have found that middle-aged women with immune-related diseases and menopausal women are at a higher risk of HILI (Tu et al., 2019a; Ge et al., 2019b). Further studies (animal experiments) on its possible mechanism showed that these may be closely related to the activation of NLRP3 inflammasomes (Gao et al., 2020; Wang et al., 2020; Gao Y. et al., 2021a). In addition, HLA-B *35:01 was found as the first specific biomarker of the HILI-susceptible population in a retrospective combined prospective observational study (Li et al., 2019).

In contrast to immune HILI, genetically HILI is closely related to single nucleotide enzyme polymorphisms (SNP) in a retrospective observational study (Wang Z. et al., 2019b; Guo et al., 2021). Drugs are detoxified in the body mainly through the phase I and phase II metabolism of the liver. The cytochrome P450 enzyme (CYP450) of phase I metabolism plays a key role, and an SNP in CYP450 due to factors such as heredity, age and gender, leads to inter-patient differences in the risk of HILI. CYP450 studies were mainly conducted using animal experiments. The expression of CYP450 enzymes is reportedly decreased in the diseased liver; in the early stage of hepatocellular carcinoma (HCC), there is a decrease in phase I metabolizing enzymes and an increase in phase II metabolizing enzymes (Braeuning and Schwarz, 2020). Therefore, HILI may be associated with abnormal drug metabolism enzymes, and inhibition of CYPs, including CYP450 isoforms 3A, 2C9, 2C19, and 2E1 in rat liver microsomes, is associated with triptolide-induced hepatotoxicity (Lu et al., 2017). Safrole, the main component of the volatile oil in Asari Radix et Rhizoma, inhibits CYP1A2 in CYP cocktail screening (Yang et al., 2018). Liver injury induced by airpotato yam consumption is closely related to the expression of CYP2E1 and CYP3A44 in the CYP450 enzyme system (Peng and Yuan, 2021).

#### 3.1.2 Indirect hepatotoxicity

In 2019, international researchers of DILI, Hoofnagle and Bjornsson (Hoofnagle and Björnsson, 2019) first proposed a third type of DILI—indirect hepatotoxicity. Compared to the previous two types of idiosyncratic DILI (IDILI), indirect hepatotoxicity, as a relatively new concept, has not been commonly accepted, and has not been described in clinical guidelines. Apart from the publications, *Drug-induced liver injury: Asia Pacific Association of Study of Liver consensus guidelines* (Devarbhavi et al., 2021), the *ACG Clinical Guideline: Diagnosis and Management of Idiosyncratic Drug-Induced Liver Injury* (Chalasani et al., 2021), the *EASL clinical practice guidelines recommendations for drug-induced liver injury in 2019* (Ofliver, 2019), nor in the *Chinese Guidelines for the management of drug-induced liver injury* and *the Guidelines for clinical diagnosis and treatment of herb-induced liver injury*, there are no respective descriptions (Yu et al., 2015; Xiao et al., 2016). Previously, this kind of toxicity was typically classified as IDILI, however, after further research; it was considered a new type of DILI. In contrast to the traditional concept of DILI, indirect hepatotoxicity is not caused by the inherent toxic effects of drugs, but by pharmacological interactions and diseases. The population segment with indirect hepatotoxicity is mainly that with pre-existing afflictions. Some experts believe that pharmacological action may change the state of the body, thereby inducing liver injury or aggravating the original liver disease. For example, certain drugs may cause liver injury by reactivating hepatitis B virus (HBV) when treating patients with HBV-associated HCC, and the concurrence of pre-existing chronic liver diseases has been associated with a worse prognosis in patients with HILI. (Hoofnagle and Björnsson, 2019; Jing et al., 2019; Gao Y. J. et al., 2021b; Xiao et al., 2021b).

### 3.2 Drug factors

#### 3.2.1 Inherent (liver) toxicity of herbs

The misconception that herbs are natural and generally non-toxic is an important factor that leads to the frequent occurrence of HILI. However, herbs cannot generally be considered non-toxic. TCM places a strong emphasis on the safety of herb, and there are records from ancient TCM literature to modern pharmacopoeias/literature. According to the safety theory of TCM, the toxicity of herbs has been recorded, and is classified as “strong poison, weak poison, common poison, and non-toxic”. China’s national health authorities have implemented strict management of toxic herbs, for example, the toxicity of herbs has been noted in various editions of the *Pharmacopoeia of the People’s Republic of China* (Peng et al., 2017; Xiao et al., 2018a), and the NMPA also refers to the risks of liver injury due to compounds such as PM and its preparations (CFDA, 2014). Herbs used as medicines can be toxic; almost no herbs are non-toxic, and the inherent potential hepatotoxicity of certain herbs should not be ignored.

#### 3.2.2 Unauthenticated herbs

In addition to inherent toxicity, unauthenticated herb quality is also a risk factor for HILI (Xiao et al., 2018a; Ge et al., 2019a). For some herbs that do not contain any or contain very few toxic ingredients, the risk of liver injury may increase because of unverified quality. There are many factors involved, including processing, mixed use of counterfeit products, storage conditions, pesticide residues, adulteration, bacterial viral contamination, and so on. This review predominantly discusses the potential risk factors in the processing of herbs, the mixed use of counterfeit products, and storage conditions.

Processing is an indispensable element before the clinical use of herbs, and it plays a key role in enhancing efficacy and reducing toxicity. However, some herbs are directly used without being processed or are processed in an irregular manner, which may increase the risk of liver injury. Using PM processing as an example, animal experiments have shown that the toxic components of PM are significantly reduced after processing (Li C. Y. et al., 2017b), and irregular processing may increase the risk of liver injury. Based on this, the difference between raw and processed PM in liver injury was further evaluated using animal experiments (Tu et al., 2015). The results showed that the biochemical indexes and pathological damage to the liver in mice treated with processed PM group were significantly lower than those in the group treated with non-processed PM, indicating that standard herb processing can significantly reduce liver injury.

The similarity in names and effects of some herbs may cause confusion. For example, Notoginseng Radix Et Rhizoma (NRR) and GS are commonly used for treating cardiovascular and cerebrovascular diseases (Ding and Xiong, 2018; Li et al., 2018). However, NRR is safe, whereas GS is more toxic and has a narrow therapeutic window. The main toxic components of GS—pyrrolizidine alkaloids, can lead to sinusoidal obstruction syndrome (SOS) and hepatic sinusoidal occlusion syndrome (HSOS). When SOS/HSOS caused by GS is not treated in a timely and reasonable manner, it frequently progresses irretrievably and rapidly to the stage of cirrhosis, or even to liver failure, according to a case study series (Li et al., 2018). Owing to the similarity of the names and efficacy of the two herbs, NRR and GS may be confused in clinical applications, leading to severe liver injury (Xiao et al., 2021a).

Safe and reasonable storage conditions are vital to ensure the quality of herbs. Inappropriate storage conditions, such as a humid environment and exposure to sunlight, etc., may cause changes in the chemical composition of herbs, resulting in higher toxicity. Animal experiments have shown that under specific lighting conditions, the “non-toxic component” -trans-stilbene glycosides of PM can be converted into “toxic components” - cis-stilbene glycosides, thereby increasing the risk of HILI (Zhang L. et al., 2017b).

### 3.3 Unreasonable prescriptions factors

#### 3.3.1 Non-conformance to traditional chinese medicine theory

Herb should be used to conforming to TCM theory; otherwise, the risk of ADRs may increase (Ge et al., 2018). The safety theory of TCM states that when the body is in a pathological state, herbs mainly target diseases (“evil”) and rarely cause injury to the body. However, when the body is healthy, a herbal treatment would not counteract a disease but instead may adversely affect the body, which may increase the risk of ADRs, including liver injury (Ge et al., 2019a). Based on this theory, animal experiments have shown that PM can induce pronounced pathological liver injury in normal rats (Tu et al., 2015), which was significantly improved in a rat model of liver fibrosis. This study fully reflects the two-way regulatory effects of the herbs. The risk of liver injury is significantly reduced under the guidance of TCM theory but is significantly increased when these principles are not followed.

#### 3.3.2 Unreasonable herb combinations

In most cases, herbs are used in the form of herbal prescriptions in clinical practice. Herbal prescriptions typically include multiple herbs. A reasonable combination of herbs can increase efficacy and reduce toxicity, whereas an unreasonable combination may increase the risk of ADRs (Ge et al., 2018; Ge et al., 2019a). Some herbs by themselves do not lead to liver injury, but their combination with other herbs may cause liver injury, possibly because interactions of components in different herbs may produce new toxic ingredients or increase the toxicity of existing ingredients. For example, Ginseng Radix Et Rhizoma (GRR) and Veratrum Nigrum (VN) alone do not cause hepatotoxicity but may do so when used together, due to the increased dissolution and absorption of veratrum alkaloids, which are toxic components of VN, and interaction with GRR, as observed using animal experiments (Ma et al., 2020). Thus, herbs combinations may lead to interactions between components, leading to a higher risk of liver injury.

#### 3.3.3 Unreasonable combination of herbs and conventional medicine

Due to unique national settings, the combination of herbal medicines and CM is very common in China. A reasonable combination can increase efficacy, however, considering the complexity of herb components, unreasonable combinations may considerably increase the risk of liver injury. Among the adverse reactions to herbal injections, the combination of herbs and CM accounted for 54.98%, as assessed using a retrospective observational study (Ge et al., 2019a). In a survey (retrospective observation) of liver injury adverse reactions related to PF preparations based on the China National Food and Drug Administration, almost half of the patients had taken a combination of herbs and CM (Ge et al., 2021). CM caffeine, pronel, and nifedipine are mainly metabolised by the metabolic enzyme CYP1A2, and the activity of CYP1A2 can be inhibited by *Astragalus membranaceus.* Their combination greatly increases the risk of liver injury (Kong et al., 2018); therefore, the risk-benefit ratio of combined treatments should be evaluated in clinical practice, rather than ignoring such risks simply to increase efficacy, especially when combining CM and herbs with a known risk of liver injury.

#### 3.3.4 Dosage and duration of treatment

The dosage and treatment duration play a key role in InHILI. When the content of herbs containing inherently toxic ingredients is low, the cumulative amount of toxic compounds does not reach the threshold for hepatotoxicity. However, when consumed in larger quantities, the cumulative amount of hepatotoxic compounds may exceed a certain threshold, and the risk of liver injury increases significantly (Xiao et al., 2018a). Therefore, according to the safety theory of TCM, herbal treatments should be initiated with low dosages, which can then be appropriately increased based on a safety assessment to minimize risks (Wang., 2004; Zhang., 2016). However, overdoses may occur from time to time in clinical practice. For example, risk factors for liver injury caused by PM and TR were investigated based on data on the NMPA, and overdose and excessive treatment duration are among the most important risk factors, based on retrospective observation (Song et al., 2019; Chalasani et al., 2021).

## 4 New progress and challenges

### 4.1 New progress

In recent years, tremendous progress has been made in HILI research, such as in the evaluation methods of HILI and IHILI-susceptible population biomarkers etc. First, at the level of laws and regulations, we have established clearer and stricter standards for the safety supervision of HILI, such as the *Pharmacopoeia of the People’s Republic of China*, the *General Principles of Clinical Research on New Chinese Medicines*, and the *Technical Guidelines for Clinical Evaluation of Herb-Induced Liver Injury* have been issued (Ge et al., 2018). Second, the clinical guidelines for HILI were released for the first time in 2016— *Guidelines for Clinical Diagnosis and Treatment of Herb-Induced Liver Injury* (Xiao et al., 2016), which provides guidelines for the clinical evaluation, diagnosis, and treatment of HILI. This guideline’s integrated evidence chain (iEC) is based on the Roussel Uclaf causality assessment method (RUCAM), which provides a scientific basis for the objective evaluation of HILI. Third, the World Health Organization-Council for International Organizations of Medical Sciences (WHO-CIOMS) and the Chinese Pharmaceutical Association Committee jointly initiated the establishment of the world’s first international cooperation alliance for the safe use of TD and issued the world’s first *Beijing Declaration on the Safe Use of Traditional Medicines*, which aimed to monitor the safety of TD (Xiao et al., 2018b). Fourth, the Chinese Society of Traditional Chinese Medicine issued the first safety guide for a single herb in 2019— *Guidelines for the Safe Use of PM* (Branch of Chinese Patent Medicine et al., 2019), which for the first time changed the research, prevention, and control of the safety (with regard to liver injury) of herbs from itself to the host, and helped improve the safety of herbs, especially regarding HILI and precision medicine (Li et al., 2019). Final, in 2020, the first HILI-focused drug safety information inquiry network “Inquiring Drug Safety” (iDS), was successfully developed and launched realizing the social mechanism of integration in HILI from the public to the clinical application (Xiao, 2020).

### 4.2 Challenges

Although HILI research has made great progress in recent years, several challenges remain. First, the pharmacoepidemiological characteristics are still unclear, which severely restricts the formulation of HILI prevention and control policies. So far, there have been no recognized epidemiological studies on HILI in China or any other country. Therefore, a high-quality HILI epidemiological study is needed to assess the incidence, demographic characteristics, the proportion of HILI among DILI cases, and prevalence of major HILI drugs, to provide high-quality evidence for the prevention and control of HILI. Second, the clinical evaluation of HILI (specific biomarkers) remains difficult. DILI evaluation mainly relies on RUCAM, but it cannot distinguish whether the origin of liver injury is a herb or CM. In response to this problem, some scholars have proposed a clinical evaluation method for HILI based on iEC. The core idea of this method is to use specific biomarkers of HILI to determine the specificity of HILI evaluation based on RUCAM. Although new potential DILI biomarkers have been discovered in recent years, such as the apoptosis index, glutamate dehydrogenase, high-mobility histone 1, and HLA-related genes, etc. (Jing et al., 2020; Chi, 2021), no biomarker specific to HILI is available. Third, the specific clinical treatments for HILI are still lacking. There are specific antidotes for treating CM-induced liver injury, such as N-acetylcysteine, which is specific to APAP (Chen H. et al., 2021b). However, there are no specific antidotes for HILI. In China and the West, HILI can result in worse outcomes than the liver injury caused by CM (Zhu et al., 2016). Therefore, it is of great importance to develop specific antidotes for HILI.

## 5 Conclusion and expectation

HDS are the main cause of DILI in Asia, especially in China, and HILI is a notoriously difficult aspect of DILI research. In the common occurrence of HILI, in addition to the inherent toxicity of herbs, there is an increasing number of non-inherent toxicity of herbs (such as immune-enhancing herbs). There are many risk factors for HILI, including three aspects: “human,” “medicine,” and “use.” The “human” factor refers to the patient’s physique, including immune status, metabolic enzymes, underlying diseases, gender, and age, etc. The “medicine” factor refers to the herb, including the unverified quality of herbs due to improper processing, use of counterfeit products, improper storage, etc. The “use” factor refers to unreasonable prescription, including non-compliance with the TCM theories, irrational combined use, and overdosing, etc.

Owing to the particular medication theory and complex composition herbs, HILI is more difficult to prevent and control than CM. We should treat HILI objectively, and herbs should not be generally assumed to be “natural” and “non-toxic”. In short, we should accelerate the construction of a HILI prevention and control system of “objective identification- mechanism analysis-risk prevention and control” based on the TCM pharmacovigilance principle of “recognizing poisons-using poisons-preventing poisons-detoxification,” and thus change its state from “not clear yet” to “preventable and controllable”.

## Glossary

AA: Aristolochic acid
ADR: adverse drug reaction
ALP: alkaline phosphatase
APAP: Acetaminophen
BF: Bruceae Fructus
CM: conventional medicine
CYP450: Cytochrome P450 enzyme
DC: Dictamni Cortex
DILI: drug-induced liver injury
DILIN: DILI network
EF: Epimedii Folium
GRR: Ginseng Radix Et Rhizoma
GS: Gynura Segetum
HBV: hepatitis B virus
HCC: hepatocellular carcinoma
HDS: herbs and dietary supplements
HILI: herb-induced liver injury
HSOS: hepatic sinusoidal occlusion syndrome
IDILI: idiosyncratic DILI
IDS: Inquiring Drug Safety
IEC: integrated evidence chain
IgA: immunoglobulin A
IHILI: idiosyncratic HILI
InHILI: intrinsic HILI
NMPA: the National Medical Products Administration of China
NRR: Notoginseng Radix Et Rhizoma
PF: Psoraleae Fructus
PM: Polygoni Multiflori Radix
RDB: Rhizoma Dioscoreae Bulbiferae
RRR: Rhei Radix Et Rhizoma
RUCAM: Roussel Uclaf causality assessment method
SNP: single nucleotide enzyme polymorphism
SOS: sinusoidal obstruction syndrome
SSH: Senecionis Scandentis Hebra
TCM: traditional Chinese medicine
TD: traditional drug
TR: Tripterygii Radix
VN: Veratrum Nigrum
WHO: World Health Organization
WHO-CIOMS: World Health Organization-Council for International Organizations of Medical Sciences.
